# Pulmonary Embolism With Syncope Treated With Inari FlowTriever®

**DOI:** 10.7759/cureus.64031

**Published:** 2024-07-07

**Authors:** Jay E Garza, Priti Shah, Napoleon Patel, Daniel Wang

**Affiliations:** 1 Dermatology, McGovern Medical School, Houston, USA; 2 Internal Medicine, McGovern Medical School, Houston, USA; 3 Emergency Department, Memorial Hermann-Texas Medical Center, Houston, USA

**Keywords:** manual aspiration thrombectomy, ctpa, inari flowtriever, pulmonary embolism with syncope, submassive pulmonary embolism

## Abstract

Pulmonary embolism (PE) is a devastating disease that can range in severity from asymptomatic to fatal. The severity and the intervention required depend on the degree of hemodynamic instability and evidence of right heart strain demonstrated on diagnostic testing. Interventions include solely anticoagulation, systemic thrombolysis, catheter-directed therapies, or surgical embolectomy depending on the severity, patient’s clinical picture, and clinician choice. Currently, there is a lack of evidence regarding which treatment is most suitable for submassive PE. This report demonstrates the benefits of aspiration thrombectomy, a catheter-directed therapy, utilizing the 24Fr Triever Aspiration Catheter (FlowTriever^® ^system;Inari Medical, Irvine, California, United States) in a 57-year-old male patient with submassive PE. The FlowTriever retrieval/aspiration system is a single-use mechanical thrombectomy device indicated for use in the peripheral vasculature and pulmonary arteries. The patient presented with syncope and concern for head trauma ultimately requiring suction embolectomy utilizing the Inari FlowTriever system. We conclude that submassive PE can be effectively treated with aspiration thrombectomy in addition to long-term anticoagulation with excellent clinical outcomes.

## Introduction

Pulmonary embolism (PE) is associated with up to 300,000 deaths annually in the United States, and longitudinal studies suggest a rising incidence [1]. Common symptoms of PE include dyspnea (82% of patients), cough (20%), and syncope (14%) [2,3]. Clinical examination may also reveal tachycardia, tachypnea, and low-grade fever [3]. Severity ranges from the majority being asymptomatic to sudden death. A high index of suspicion should be maintained so that patients can be rapidly identified and started on appropriate treatment [2,3]. 

Interventions include solely anticoagulation, systemic thrombolysis, catheter-directed therapies, or surgical embolectomy depending on the severity, patient’s clinical picture, and clinician choice. Anticoagulation is typically all that is necessary for patients in the low-risk category. Increasing the intensity of intervention, systemic thrombolysis can be utilized on patients who qualify for intermediate risk or submassive PE. Systemic thrombolysis has been shown to be effective for patients with a low risk of bleeding [4]. Absolute contraindications include but are not limited to a history of hemorrhagic stroke, ischemic stroke within the last six months, and head injury or trauma within the last three weeks. In those with absolute contraindications to systemic thrombolysis, catheter-directed therapy has been shown in preliminary studies to be an excellent treatment choice [1,5]. 

This report demonstrates the benefits of aspiration thrombectomy, a catheter-directed therapy, utilizing the 24Fr Triever Aspiration Catheter (FlowTriever® system; Inari Medical, Irvine, California, United States) in a patient with submassive PE with concern for head trauma. The device itself consists of a large lumen catheter which is connected to a syringe. The catheter lumen allows the aspiration of the majority of the clot via negative pressure generated by the syringe. It then allows the passage of self-expanding nitinol mesh that forms three disks to capture and remove any remaining thrombus.

## Case presentation

Our patient was a 57-year-old male with a past medical history of gout who presented to the emergency room with three days of bilateral knee swelling and pain that was associated with recurrent falls one month prior to admission. Initial hospital evaluation was directed at the left knee where magnetic resonance imaging revealed complete rupture of the proximal patellar tendon, meniscal tears, and joint effusion with synovitis. Three days into hospitalization while on heparin 5000 units every eight hours for deep venous thrombosis prophylaxis, he experienced near syncope during physical therapy (PT). Two days later he experienced a full syncopal episode while working with PT and suffered a head injury. He was tachycardic but normotensive (Table 1).

**Table 1:** Vitals at admission, during the event and escalation to ICU, and post thrombectomy *Vapotherm is a high-flow nasal cannula device that can provide additional respiratory support (Vapotherm Inc., Exeter, New Hampshire, United States)

	Initial	Event	Post-treatment 24 hours
				Systolic Blood Pressure (mmHg)	139	114	135
Heart Rate (beats per minute)	90	112	85
Oxygen Saturation (%)	96	93	95
Oxygen Delivery Modality	Room air	Vapotherm* 20L/60%	Nasal Cannula 2L
Temperature (^o^F)	98.8	98.3	98.3

**Figure 1:**
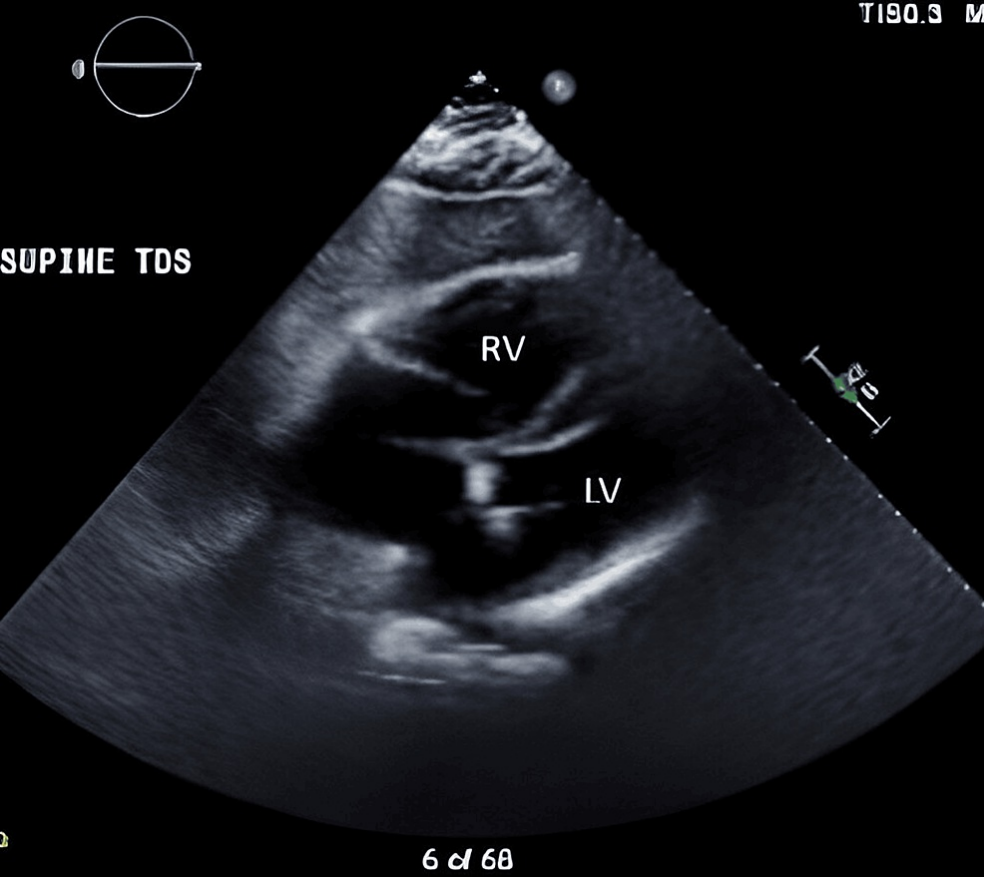
Echocardiography while the patient was supine Left ventricle appears normal in size with low normal systolic function; right ventricle is dilated with reduced function

**Figure 2:**
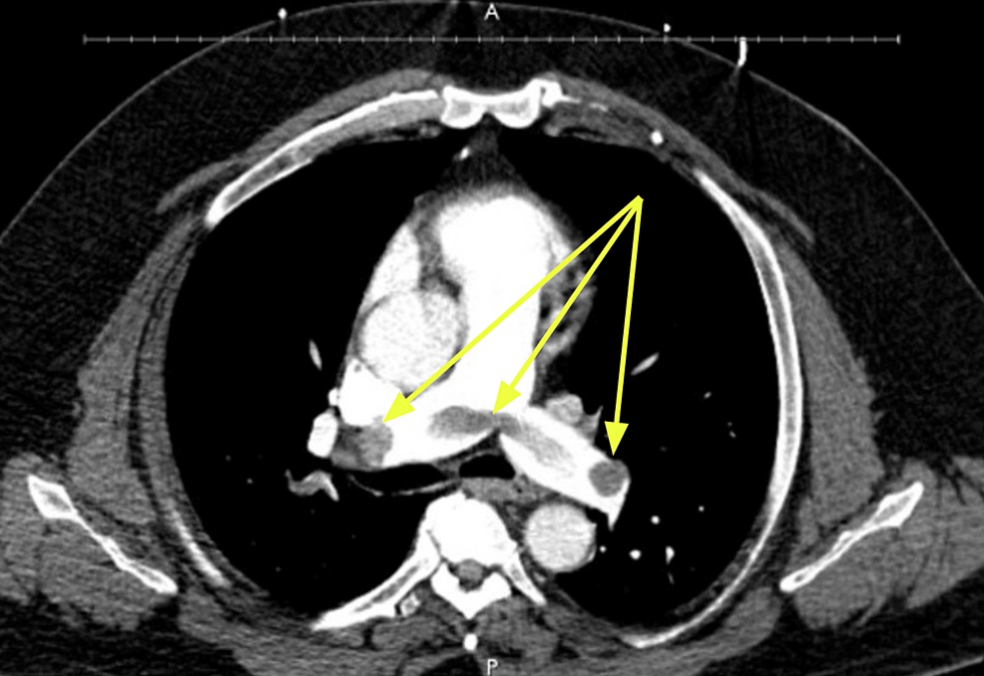
Chest computed tomography angiography Arrows pointing toward hypodensities within the bilateral pulmonary arteries are indicative of a bilateral pulmonary embolism

The patient's oxygen requirements were elevating, his troponin I peaked at 509 pg/mL, and B-type natriuretic peptide was elevated to 247 pg/mL. Echocardiogram (ECHO) revealed a low normal systolic function with right ventricular dilation and reduced function (Figure 1). A computed tomography angiography (CTA) of the chest was urgently completed, which showed large, extensive central and peripheral bilateral pulmonary emboli (Figure 2).

**Figure 3:**
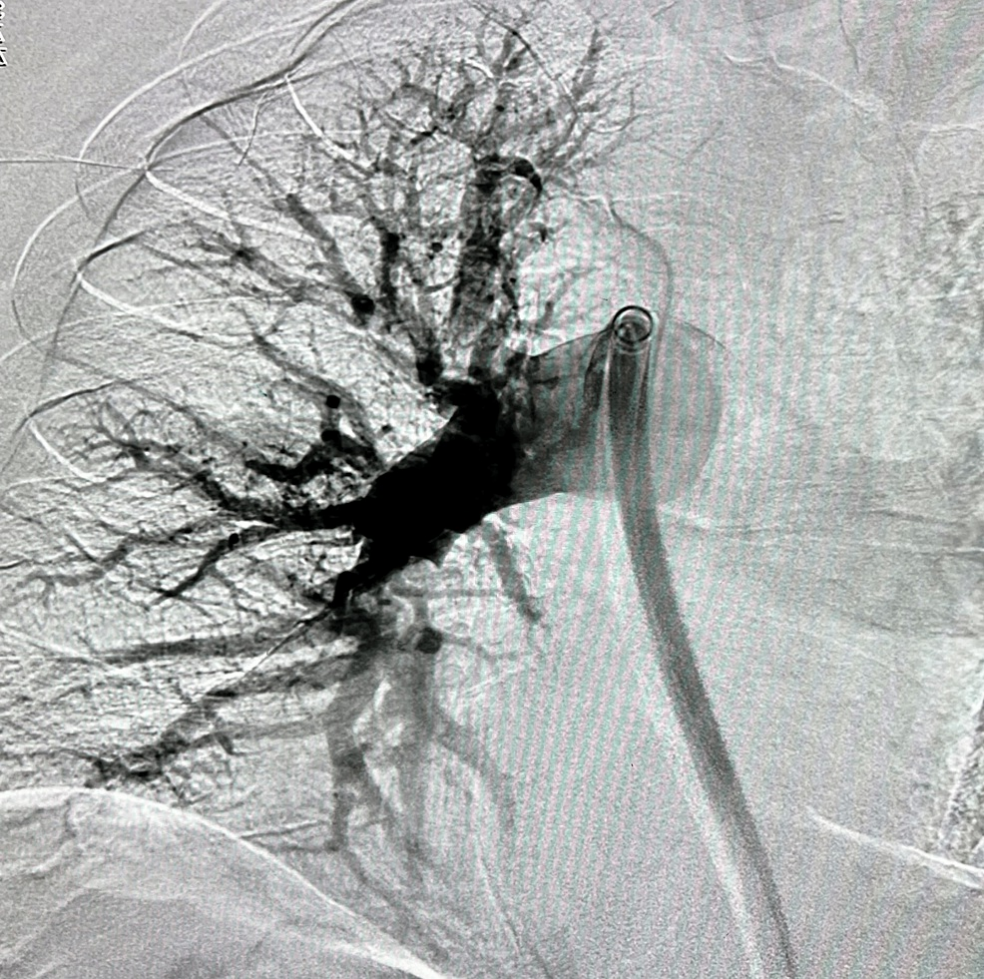
Fluoroscopy of right lung during mechanical thrombectomy Right lung clot burden visualized using the FlowTriever® system (Inari Medical, Irvine, California, United States); right pulmonary artery distension 3.2 cm.

**Figure 4:**
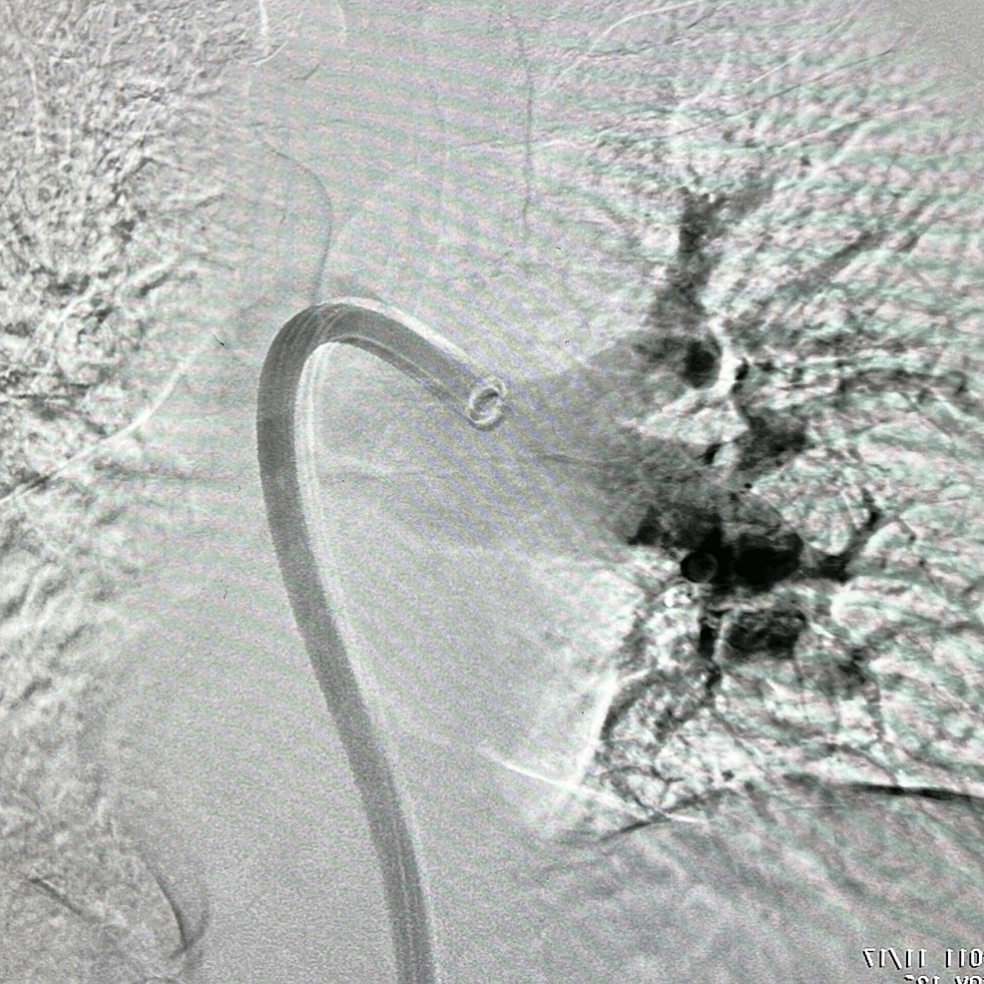
Fluoroscopy of left lung during mechanical thrombectomy Left lung clot burden visualized using the FlowTriever® system (Inari Medical, Irvine, California, United States). Left pulmonary artery distension 3.2 cm.

**Figure 5:**
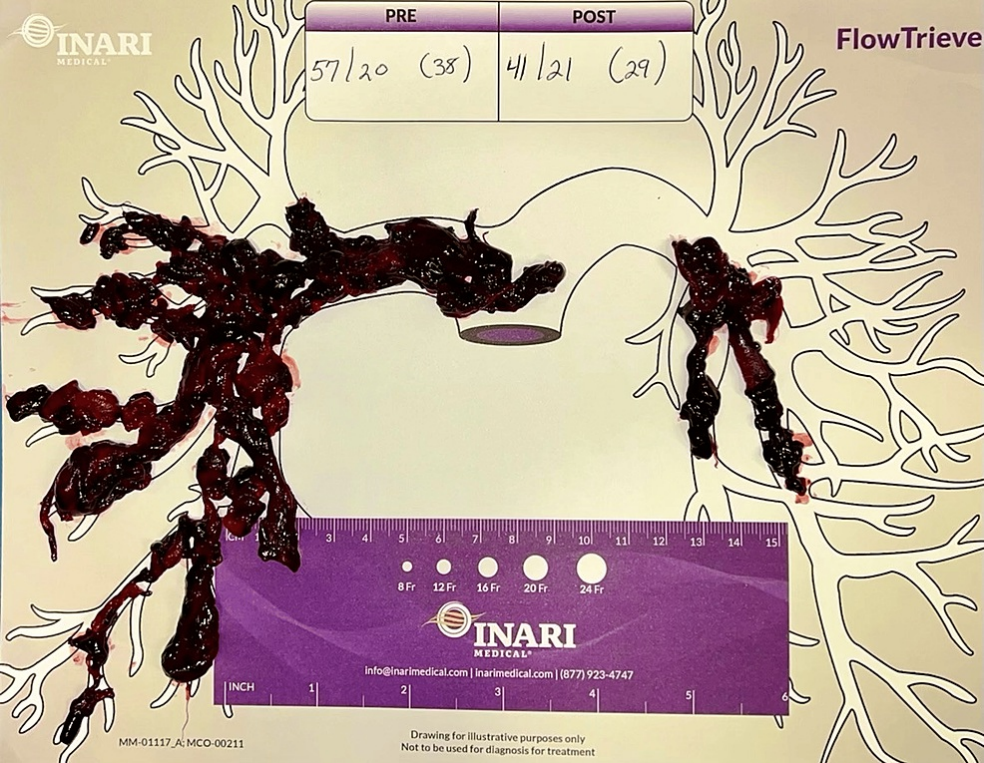
Inari FlowTriever® lung map Clots removed and mapped out on the Inari FlowTriever® (Inari Medical, Irvine, California, United States) lung map displaying improvement in pressures from before and after clot removal.

**Figure 6:**
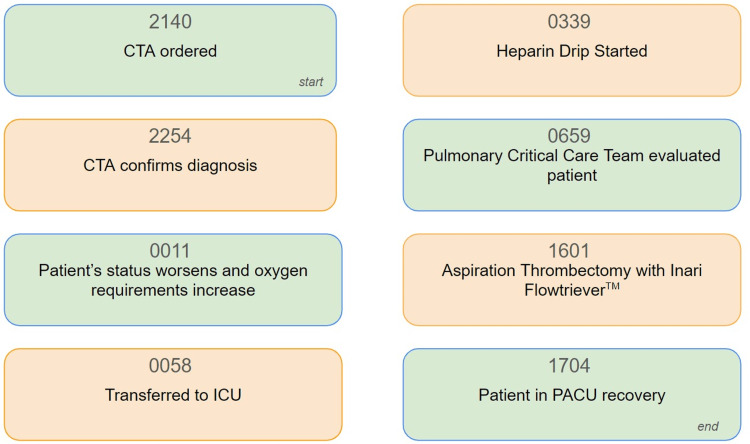
Timeline of events from ordering confirmatory imaging to completion of procedure CTA: computed tomography angiography; ICU: intensive care unit; PACU: post-anesthesia care unit

A saddle pulmonary embolism was visualized crossing the main right and left pulmonary arteries extending into all lobes of both lungs down to the subsegmental levels. Lower extremity venous Doppler revealed occlusive thrombosis of the distal right popliteal vein and nonocclusive thrombosis of the proximal, right popliteal vein. Verbal and written consent was obtained from the patient, and he was started on a heparin infusion for initial management. Because the patient’s oxygen requirement rapidly escalated to high-flow nasal cannula (HFNC) he was transferred to the intensive care unit (ICU) (Table 1). Due to the patient’s clot burden along with his clinical decline, he was urgently taken for right heart catheterization and suction embolectomy utilizing the 24Fr Triever Aspiration Catheter (Figures 3, 4) [6]. The procedure was completed successfully without complications, and the mean pulmonary arterial pressure (PAP) decreased from 38 mmHg to 29 mmHg (Figure 5). He was quickly weaned to room air, and his anticoagulation was switched from heparin infusion to apixaban. Figure 6 details the order of events from ordering confirmatory imaging to completing the procedure. He was discharged in stable condition to a rehabilitation facility.

## Discussion

The annual incidence of PE in North America ranges from 0.75 to 2.69 cases per 1000 individuals [7]. The actual figures may be substantially higher due to the silent development of PE in patients with deep vein thrombosis [2,3]. Despite ranking third among cardiovascular diseases, following coronary artery disease and stroke, PE imposes a significant mortality risk, with untreated cases associated with rates as high as 30% [2,3]. However, prompt diagnosis and treatment substantially reduce mortality to 8%, highlighting the critical importance of early and appropriate intervention [2,3,5,8]. Moreover, the acute nature of PE underscores its severity, with up to 10% of patients succumbing suddenly, two-thirds of whom do so within two hours of presentation [2]. These statistics emphasize the urgent need for improved awareness, detection, and management strategies to mitigate the impact of PE and venous thrombosis on public health [2].

The classification of severity differs slightly between the American Heart Association (AHA) and European Society of Cardiology (ESC) classifications. The degree of severity begins with low-risk PE. These patients likely have some clinical signs, and imaging may be suggestive of the diagnosis. However, these patients don’t qualify for submassive or intermediate-low risk classification because there is no right heart strain or injury on diagnostic testing nor do they have 1 point on the simplified Pulmonary Embolism Severity Index (sPESI) score (age >80 years, cancer, chronic heart failure or pulmonary disease, heart rate ≥ 110, systolic blood pressure < 100, arterial partial pressure of oxygen (PaO2) <90%) [9].

The next classification is submassive or intermediate risk on the AHA or ESC classification, respectively [4,10]. This classification is where the largest contrast between AHA and ESC lies. According to the AHA, the submassive classification belongs to patients with right ventricular strain on either imaging or blood tests [10]. Right ventricular strain on imaging is demonstrated by a right/left ventricular ratio > 0.9 on computed tomography pulmonary angiography or ECHO while right heart strain detected by blood tests includes elevated troponins or elevated B-type natriuretic peptide (BNP) [4,10]. The ESC criteria for intermediate-risk PE are broader with two subgroups that include patients who have an sPESI score ≥1. The ESC then subdivides intermediate-risk patients into those that have one or no findings of RV strain and those that have both imaging and diagnostic testing consistent with right ventricular strain. The most severe classification is massive or high risk which is characterized by hemodynamic instability that is demonstrated by hypotension < 90 mmHg, a drop of > 40 mmHg, or a need for vasopressor support [10]. 

PE can present with a wide variety of symptoms and severity which necessitate the use of imaging to confirm diagnosis [8,9]. ECHO has been widely used as a screening tool for pulmonary embolism due to its accessibility and ease of use [9]. Fields et al. showcase that ECHO is actually better utilized to rule in PE, but they claim that ECHO parameters do not have the capability to rule PE out [10]. In their report, they explored multiple ECHO parameters to better determine which may be more sensitive. They found that right ventricular end-diastolic dilation seems to have the highest sensitivity (83%) which is similar to the claims made for CTA [5,8,9].

CTA is the most common way to detect a pulmonary embolism in the United States [8]. CTA has been described to have a high sensitivity (83%) and specificity (96%) [8]. When combined with clinical probability scoring such as the PESI index or sPESI, the positive predictive value can reach up to 96% for high-risk PE and 92% for intermediate-risk PE [8]. The transthoracic echocardiography in our case was first used because of convenience, but it was quickly followed by CTA. CTA in this case was utilized because it is minimally invasive, relatively cost-effective, and has a high diagnostic capability to rule in PE.

Koyabashi et al. clarified trends in both in-hospital mortality rates and treatment patterns among the risk stratification of PE. Regarding treatment patterns, high-risk PE patients were more likely to undergo advanced therapies such as systemic thrombolytics, surgical embolectomy, and mechanical circulatory support compared to their intermediate-risk counterparts (41.9% vs 30.2%; P < 0.001) [11]. High-risk patients had a higher in-hospital mortality (20.6% vs 3.7%; P < 0.001) and a chance of major bleeding (10.5% vs. 3.5%; P < 0.001). These findings highlight the significant impact of risk stratification on treatment decisions and outcomes in PE patients. This review also emphasizes the benefit of advanced therapies within the intermediate-risk PE group [11].

Furthermore, current guidelines recommend catheter-based mechanical thrombectomy only for hypotensive patients who have high bleeding risk, failed thrombolysis, or shock [5,11]. Mechanical thrombectomy with the Inari FlowTriever device has surfaced as a safe and effective alternative and adjunct advanced therapy for PE [5]. The FlowTriever retrieval/aspiration system is a single-use mechanical thrombectomy device indicated for use in the peripheral vasculature and pulmonary arteries. The device consists of a large lumen catheter, containing a mesh, which is then connected to a syringe to quickly remove large volumes of clots. The catheter lumen allows the passage of self-expanding nitinol mesh that forms three disks to capture, remove, and deliver the clot to the syringe via aspiration. It received United States Food and Drug Administration 510(k) clearance for the PE indication in May 2018 [5]. Since then, multiple studies, the most prominent of which is FlowTriever Pulmonary Embolectomy Clinical Study (FLARE), showcase its benefits and drawbacks. First, it can potentially more quickly achieve hemodynamic stability than traditional anticoagulation. Indeed, in the present study, the patient presenting with pulmonary hypertension achieved a significant decrease in PA pressure immediately following thrombectomy. The Inari FlowTriever may also be used in patients with a high risk for bleeding, and could potentially decrease the length of stay in the hospital [5]. 

Fortunately, our patient did not suffer complications from this procedure, but the potential risk is, of course, bleeding. Intracerebral hemorrhage, although rare, has been reported along with rates as low as 9.3% for major bleeding [9]. More studies are required to further determine which groups of patients would most benefit from this intervention considering the robust benefits and ever-present risks.

## Conclusions

This patient's clinical presentation with recurrent syncope and hemodynamic instability prompted the investigation for a pulmonary embolism. This case demonstrates how FlowTriever could benefit patients with submassive PE; however, further studies are needed to better clarify patient selection and timing of procedure to best maximize the benefits of the device.
